# Transplanum polare approach to the anterior mesiotemporal region

**DOI:** 10.1007/s00701-025-06530-7

**Published:** 2025-04-22

**Authors:** Daniele Starnoni, Lorenzo Giammattei, Roy T. Daniel, Pablo González-López

**Affiliations:** 1 Department of Clinical Neurosciences, Neurosurgery Service and Gamma Knife Center, Centre Hospitalier Universitaire Vaudois (CHUV), Lausanne, Switzerland; 2 Faculty of Biology and Medicine (FBM), University of Lausanne (UNIL), Lausanne, Switzerland; 3 Department of Neurosurgery, Hospital General Universitario de Alicante. Department of Optics, Pharmacology & Anatomy, University of Alicante, Alicante, Spain

**Keywords:** Mesiotemporal lesions, Planum polare, Sylvian fissure, Amygdalo-hippocampectomy

## Abstract

**Background:**

The transplanum polare approach is a surgical technique targeting mesiotemporal structures, such as the amygdala and hippocampus. It offers a safer alternative to transcortical and transsylvian routes, reducing risks to white matter tracts, optic radiations, and vascular structures.

**Method:**

This method involves a pterional craniotomy with tailored sylvian fissure dissection to access the planum polare. Dynamic retraction, image guidance, and subpial dissection ensure precise resection while preserving critical neural and vascular anatomy.

**Conclusion:**

By minimizing disruption and technical complexity, the transplanum polare approach enhances safety and efficacy, reducing complications and improving outcomes for patients with mesiotemporal lesions.

**Supplementary Information:**

The online version contains supplementary material available at 10.1007/s00701-025-06530-7.

## Relevant surgical anatomy

The sylvian fissure is formed by the infolding of the frontal, parietal, and temporal opercula over the insula. The limen insulae, composed of uncinate fasciculus fibers covered by gray matter, appears as an arched ridge extending from the anterior long gyrus to the posterior orbital gyrus. The uncinate fasciculus, originates in the frontoorbital and inferolateral frontal lobe, passing under the anterior limiting sulcus to the temporal pole. Superior and dorsal to the uncinate fasciculus lies the inferior frontooccipital fasciculus, which connects the inferior frontal gyrus and frontoorbital lobe, to the posterior temporal, parietal, and occipital lobes [4, 5, 7].

The temporal operculum, forming the inferior sylvian fissure wall, includes the planum temporale, Heschl’s gyrus, and planum polare. The planum polare, a depression on the anterior temporal lobe’s superior surface, lies between Heschl’s gyrus and the uncus. It is divided into an anteroposteriorly oriented part and a lateromedially deviating section, separated by the rhinal incisura from the uncus. The superior uncus corresponds to the medial nucleus of the amygdala, while the temporal horn projects below the insula's inferior limiting sulcus [4, 7].

The medial deviation of the planum polare and retraction of the pars triangularis create a significant lateral sylvian fissure space. The middle cerebral artery (MCA) is divided into four segments: M1 extends from the carotid bifurcation to the limen insulae; M2 courses around the insular pole to the superior and inferior limiting sulci; M3 follows the frontal, parietal, and temporal opercula, curving over the planum polare; and M4 constitutes the cortical branches. The planum polare depression accommodates the M3 segment's curves.

## Description of the technique

The patient is positioned supine with the head rotated 15–20° toward the opposite side and slightly extended. This alignment orients the sylvian fissure vertically, minimizing the need for retraction during dissection. The surgical incision begins at the upper border of the zygomatic arch near the tragus and extends posteriorly along the hairline to the midline. To safeguard the frontal branch of the facial nerve, a subgaleal and interfascial dissection is carried out until the orbital rim and temporal muscle are exposed. Following the temporal muscle dissection, a pterional craniotomy is performed, centered on the sylvian fissure. A standard C-shaped dural opening, focused on the fissure, provides adequate exposure of the frontal and temporal opercula.

The sylvian fissure is opened from distal to proximal, starting at the pars opercularis of the inferior frontal gyrus. The dissection utilizes alternating sharp and blunt techniques under high magnification, employing the inside-to-outside approach. Retractors are avoided; instead, a suction tube serves as a dynamic retractor, while veins are gently displaced temporally when feasible. The dissection continues until the frontal and temporal lobes are separated. The early dorsolateral branches of the M1 segment are meticulously examined, with the temporopolar, anterior temporal, and occasional uncal arteries carefully identified. If required, these vessels are delicately separated from the mesial temporal lobe and retracted medially using cottonoids. The M3 branches of the MCA are mobilized medially to visualize the anterior and inferior portions of the insular limiting sulcus, the limen insulae, and the planum polare. Unlike the transylvian transinsular approach, this technique avoids dividing small vessels entering the insula, and do not necessarily requires to dissect the fissure till reaching the MCA bifurcation requiring exposure only to the extent necessary to reach the planum polare.

**Fig. 1:**
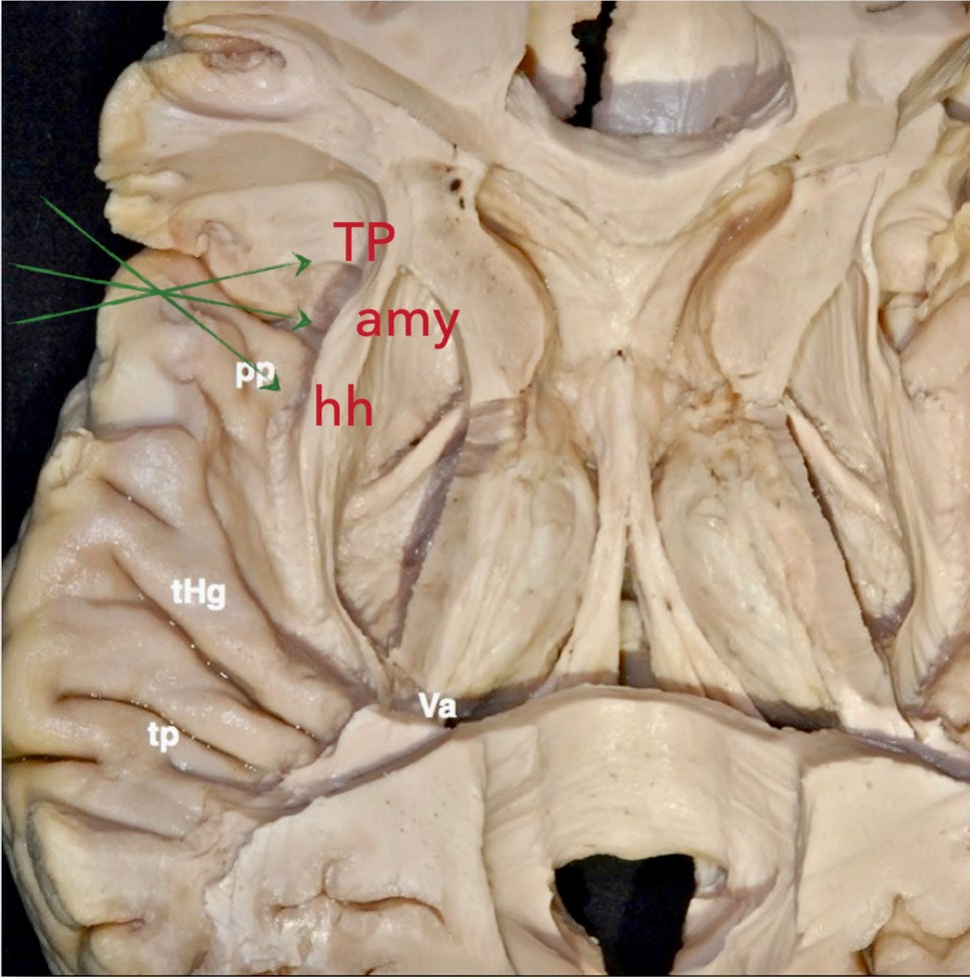
Superior view of the left temporal lobe’s upper surface. The planum temporale (TP) typically consists of two transverse gyri, located just behind Heschl’s anterior transverse temporal gyrus, and oriented toward the ventricular atrium (Va). The planum polare forms the superior surface of the anterior temporal lobe, extending between Heschl’s gyrus posteriorly and the uncus anteromedially. Green arrows indicate the anteroposterior extent of the surgical exposure via the transplanum polare approach, which provides access to mesiotemporal structures, including the temporal pole (TP), amygdala (Amy), anterior hippocampus (Hh), and parahippocampal gyrus

A small corticotomy, approximately 1 cm, is created at the planum polare in its anteromedially oriented section, located between Heschl’s gyrus and the rhinal sulcus (Fig. 1). Image-guidance systems or intraoperative ultrasound assist in directing the dissection toward the amygdala and the hippocampal head. The white matter is carefully dissected to expose the amygdala, and posteromedial dissection enables access to the anterior temporal horn through it antero-medial wall preserving Meyer’s loop fibers. Once the ventricular anatomy, including the hippocampal head and choroid plexus, is identified, the lesion is resected. The surgical field is maintained along an anteroposterior axis parallel to the choroidal fissure but directed in a superoinferior trajectory (Fig. 1). This prevents breaching the temporal horn roof or damaging the temporal stem avoiding the optic radiation and the inferior fronto-occipital fasciculus. Closure follows standard protocols for the pterional approach.

## Indication

**Fig. 2:**
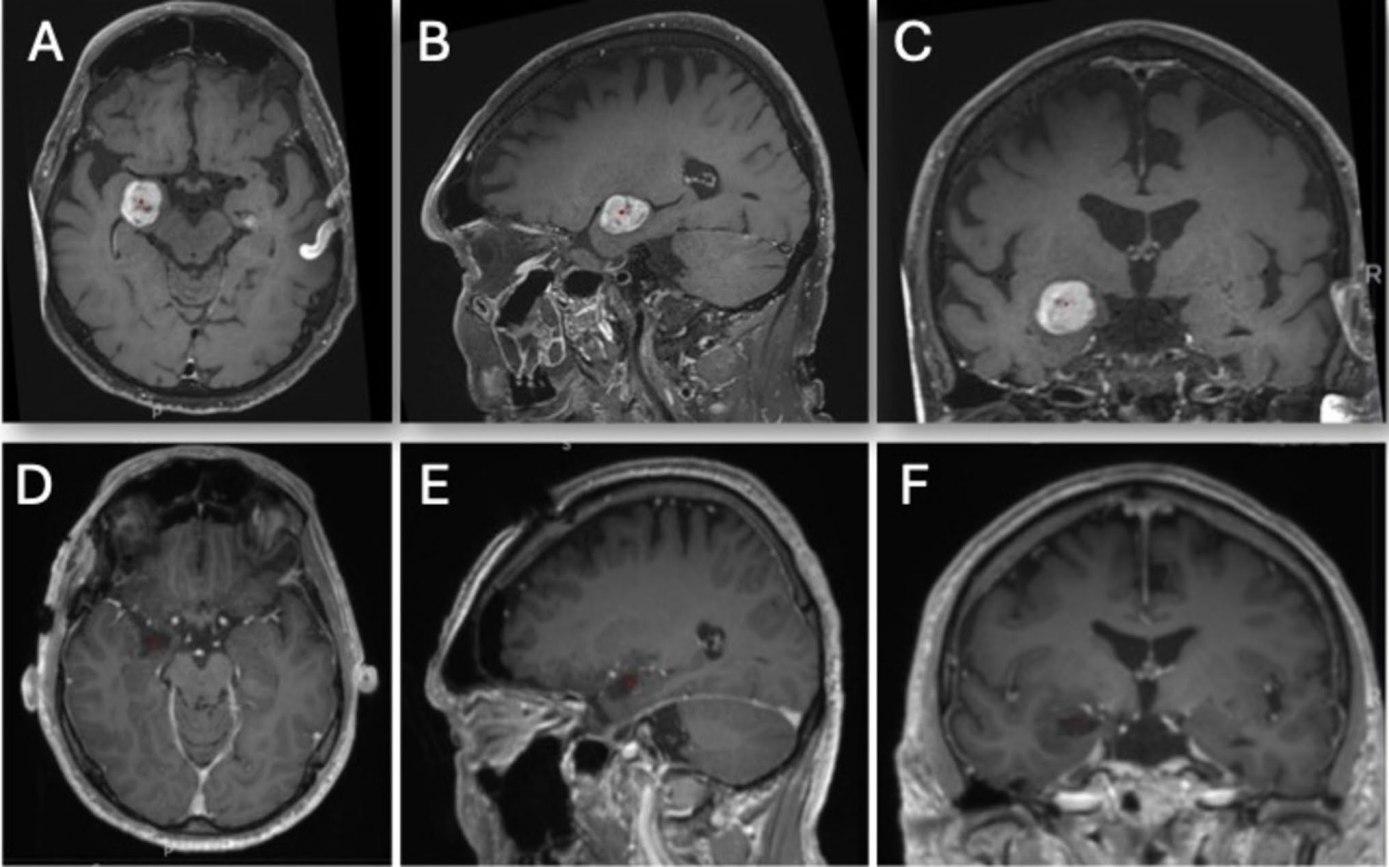
**A**–**C** Preoperative gadolinium-enhanced T1-weighted axial, sagittal, and coronal MR images depicting a well-defined, round, enhancing lesion within the amygdala. **D**–**F** Postoperative gadolinium-enhanced T1-weighted axial, sagittal, and coronal MR images demonstrating gross total resection with no evidence of neocortical disruption

This approach is ideal for lesions in the mesiotemporal region, including the amygdala, anterior hippocampus, and parahippocampal gyrus (Fig. 2). It is also suitable for selective amygdalo-hippocampectomy in patients with medically refractory complex partial epilepsy where the epileptogenic focus lies within or near the mesial temporal lobe.

Various approaches to the mesial temporal lobe exist [1–3, 6, 8]. Transcortical-transventricular and transylvian-transventricular techniques rely on unroofing the temporal horn to access mesial temporal structures. The transcortical route through the middle temporal gyrus risks damaging lateral cortex and disrupting white matter tracts, such as the inferior, middle, and fusiform gyri. The transsylvian approach avoids lateral damage but risks injuring optic radiations and the inferior fronto-occipital fasciculus (IFOF), leading to visual field deficits or cognitive impairments. Furthermore, deeper dissection of the sylvian fissure increases technical complexity and the risk of vascular injury.

**Fig. 3:**
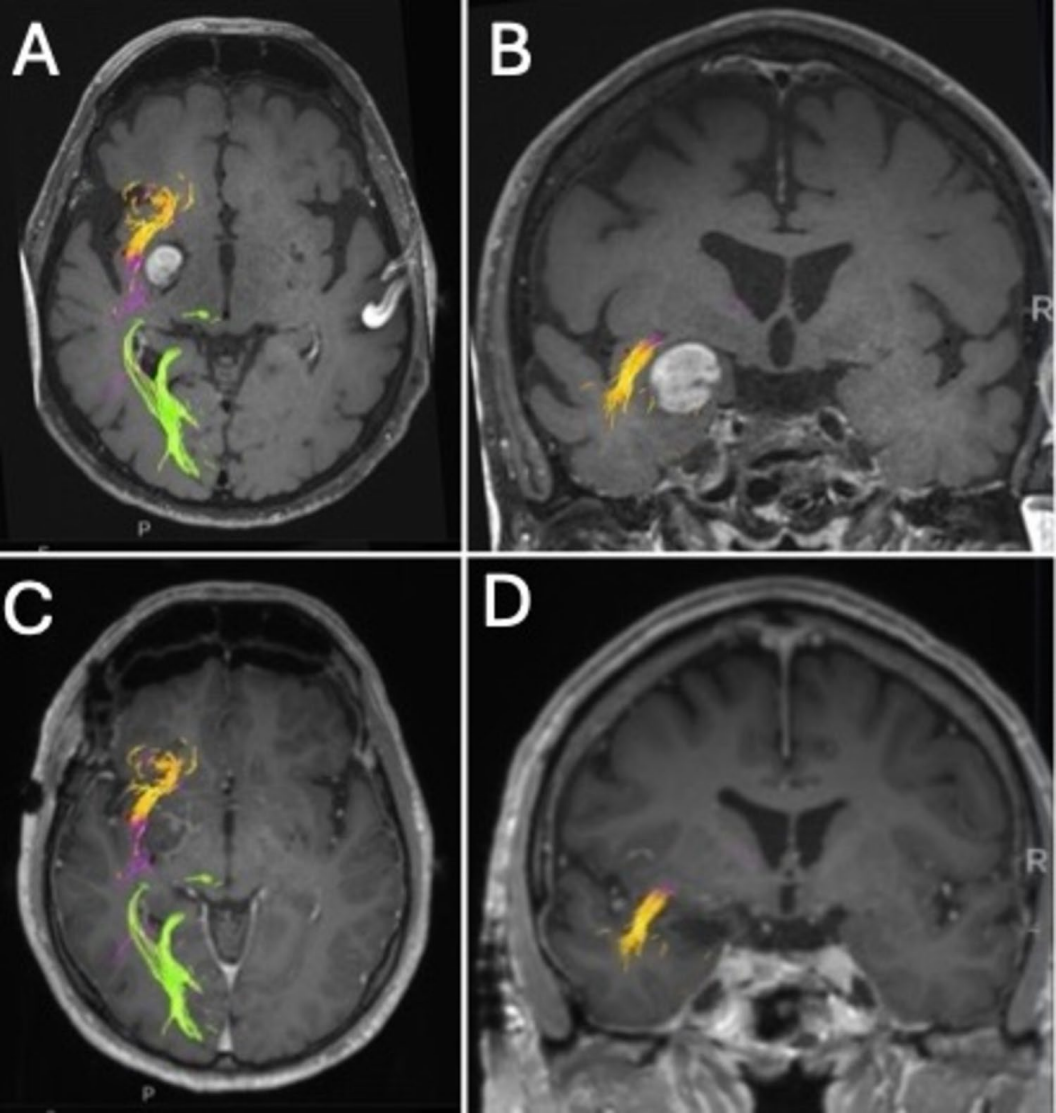
**A**, **B** Preoperative gadolinium-enhanced T1-weighted axial, sagittal, and coronal MR images illustrating the tumor’s relationship with white matter tracts. The lesion is situated anteromedial to the inferior fronto-occipital fasciculus (IFOF, purple fibers) and medial to the uncinate fasciculus (yellow fibers). **C**, **D** Postoperative images demonstrate preservation of these fiber tracts. The optic radiations (green fibers) are positioned more posteriosuperiorly

The transplanum polare approach minimizes these risks by requiring only limited sylvian fissure dissection to reach the planum polare, simplifying the procedure and reducing vascular injury (Fig. 3). Subtemporal approaches, while preserving the lateral temporal lobe, involve a circuitous trajectory requiring substantial retraction. This increases the risk of injury to the vein of Labbé and damage to the inferior temporal and fusiform gyri, especially in the language-dominant hemisphere, where the temporobasal language area is located. The transplanum polare approach minimizes anatomical disruption, offering a safer and more efficient route to mesiotemporal lesions.

## Limitations

This approach provides a confined working window, particularly for large lesions extending posteriorly or infiltrating the temporal stem. Access to the posterior mesiotemporal region is generally limited to the midmesencephalic level, complicating complete visualization and resection. Additionally, anatomical variations in the sylvian fissure can pose challenges during dissection.

## How to avoid complications

Preoperative tractography is essential for mapping white matter tracts, especially in large lesions. Broad anteroposterior splitting of the sylvian fissure minimizes retraction on the temporal and frontal opercula, providing clear anatomical visualization. Dynamic retraction techniques should replace fixed retractors to reduce tissue injury.

Access to the temporal horn is achieved via the anteromedial ventricular wall, avoiding the ventricular ceiling to preserve critical white matter bundles. Subpial dissection under constant cisternal control minimizes the risk of damaging lenticulostriate and perforating vessels. Intraoperative navigation or ultrasound ensures precise lesion localization, reducing disruption to white matter pathways. Neurophysiological monitoring helps protect functional pathways, minimizing neurological deficits.

## Specific information to give to the patient about surgery and potential risks

The risk of visual impairment, such as homonymous superior quadrantanopia, is 3–5%. We systematically assess the visual field preoperatively using automated perimetry to establish a baseline, with postoperative testing performed initially and repeated at 3 months only if deficits are noted. Damage to the IFOF near the temporal stem or roof of the temporal horn may cause language or cognitive disturbances in 5% of cases. Neurocognitive function is also evaluated pre- and postoperatively through standardized assessments, focusing on memory, language, and executive function, with follow-up at 1 week, 3 months, 6 months, and 1 year. Rare sensory-motor or language deficits may occur due to vascular injury or perforator avulsion. Additional risks include infection, bleeding, cerebrospinal fluid leakage, or postoperative seizures. Temporary deficits, such as mild speech or motor impairments, may also occur but often resolve. Thorough planning and postoperative care focus on minimizing these risks and optimizing long-term functional outcomes.

## 10 key points summary

1. The transplanum polare approach is optimal for accessing mesiotemporal lesions, particularly the amygdala, anterior hippocampus, and parahippocampal gyrus.
2. This approach is effective for resecting tumors and performing selective amygdalo-hippocampectomy in cases of medically intractable epilepsy.
3. A thorough understanding of the sylvian fissure, mesiotemporal structures, and key white matter tracts, such as the inferior fronto-occipital fasciculus (IFOF) and optic radiation, is crucial to minimize complications.
4. A wide opening of the sylvian fissure, and targeted corticotomy of the planum polare for access to mesiotemporal structures.
5. Preoperative tractography aids in planning.
6. Broad sylvian fissure splitting, dynamic retraction, and precise subpial dissection help minimize retraction injuries and protect critical vascular and white matter structures.
7. Aggressive manipulation of vessels should be avoided. Thorough dissection and gentle handling are essential to prevent traction, spasm, or vascular injury.
8. This technique avoids many of the limitations of transcortical, transsylvian, and subtemporal approaches, such as damage to the optic radiation and the inferior fronto-occipital fasciculus (IFOF). It also reduces the likelihood of inadvertently entering the temporal stem and provides direct cisternal control over mesiotemporal structures
9. The risk of homonymous superior quadrantanopia is 3–5%. Injury to the IFOF may cause language or cognitive disturbances, particularly in the dominant hemisphere.
10. Clear communication about potential risks, including visual deficits, temporary neurological impairments, and rare vascular injuries, is essential.

## Supplementary Information

Below is the link to the electronic supplementary material.

Supplementary file1 (MOV 434636 KB)

## Data Availability

No datasets were generated or analysed during the current study.

## References

[1] Campero A, Troccoli G, Martins C, Fernandez-Miranda JC, Yasuda A, Rhoton AL Jr (2006) Microsurgical approaches to the medial temporal region: an anatomical study. Neurosurgery 59:ONS279–307. 10.1227/01.NEU.0000223509.21474.2E. discussion ONS307-278 17041498 10.1227/01.NEU.0000223509.21474.2E

[2] Choi C, Rubino PA, Fernandez-Miranda JC, Abe H, Rhoton AL Jr (2006) Meyer’s loop and the optic radiations in the transsylvian approach to the mediobasal temporal lobe. Neurosurgery 59:ONS228–235. 10.1227/01.NEU.0000223374.69144.81. discussion ONS235-226 17041492 10.1227/01.NEU.0000223374.69144.81

[3] Faust K, Schmiedek P, Vajkoczy P (2014) Approaches to temporal lobe lesions: a proposal for classification. Acta Neurochir (Wien) 156:409–413. 10.1007/s00701-013-1917-4 24201756 10.1007/s00701-013-1917-4

[4] Panesar SS, Yeh FC, Deibert CP, Fernandes-Cabral D, Rowthu V, Celtikci P, Celtikci E, Hula WD, Pathak S, Fernandez-Miranda JC (2017) A diffusion spectrum imaging-based tractographic study into the anatomical subdivision and cortical connectivity of the ventral external capsule: uncinate and inferior fronto-occipital fascicles. Neuroradiology 59:971–987. 10.1007/s00234-017-1874-3 28721443 10.1007/s00234-017-1874-3

[5] Ribas EC, Yagmurlu K, Wen HT, Rhoton AL Jr (2015) Microsurgical anatomy of the inferior limiting insular sulcus and the temporal stem. J Neurosurg 122:1263–1273. 10.3171/2014.10.JNS141194 25859806 10.3171/2014.10.JNS141194

[6] Ture U, Harput MV, Kaya AH, Baimedi P, Firat Z, Ture H, Bingol CA (2012) The paramedian supracerebellar-transtentorial approach to the entire length of the mediobasal temporal region: an anatomical and clinical study. Laboratory investigation. J Neurosurg 116:773–791. 10.3171/2011.12.JNS11791 22264179 10.3171/2011.12.JNS11791

[7] Wen HT, Rhoton AL Jr, de Oliveira E, Castro LH, Figueiredo EG, Teixeira MJ (2009) Microsurgical anatomy of the temporal lobe: part 2--sylvian fissure region and its clinical application. Neurosurgery 65:1–35. 10.1227/01.NEU.0000336314.20759.85 19934983 10.1227/01.NEU.0000336314.20759.85

[8] Yasargil MG, Krayenbuhl N, Roth P, Hsu SP, Yasargil DC (2010) The selective amygdalohippocampectomy for intractable temporal limbic seizures. J Neurosurg 112:168–185. 10.3171/2008.12.JNS081112 19575575 10.3171/2008.12.JNS081112

